# Cross-cultural Validation of the Health Mindset Scale for Brazil

**DOI:** 10.1055/s-0044-1779329

**Published:** 2024-04-10

**Authors:** Brunno Nóbrega Queiroga, Thiago Batista Ravanelli, Lucas Vendas Maluf Braga, Murilo Alexandre, Rodrigo Góes Medea de Mendonça, Robert Meves

**Affiliations:** 1 Departamento de Ortopedia e Traumatologia, “Pavilhão Fernandinho Simonsen”, Faculdade de Ciências Médicas da Santa Casa de São Paulo (FCMSCSP), São Paulo, SP, Brasil

**Keywords:** quality of life, surveys and questionnaires, reproducibility of the tests, health

## Abstract

**Objective** To cross-culturally validate the Health Mindset Scale for Brazil, as well as adapt the terms and questionnaires for adequate understanding of Brazilians, using factor analysis as an instrument to validate its reliability.

**Methods** Cross-cultural validation of the Health Mindset Scale into Brazilian Portuguese using the Beaton method, Cronbach's alpha calculation and factor analysis

**Results** The sample consisted of 215 patients aged between 18 and 87 years (M = 41.98; SD = 15.72), with a mean age between 31 and 50 years (42.0%), and female (52 .6%), a marginally significant difference (p < 0.10) between men and women was observed for item 3. In this item, men's mean (M = 5.48; SD = 0.99) was higher than the average for women (M = 5.10; SD = 1.22), with a small effect size for the difference (d = 0.26). Pearson's r correlation coefficient was examined between the mean score and age (r = −0.21; p = 0.002), the result of which indicated a weak, negative and significant linear relationship. The older the age, the lower the average score on the Health Mindset Scale.

**Conclusion** The version of the health-focused mindset scale for Brazilian Portuguese was introduced and cross-culturally validated, demonstrating good reliability with a Cronbach's alpha of 0.786. Consequently, it constitutes a new instrument for clinical practice and can be correlated with established scales in the literature.

## Introduction

Mindset theory proposes the ways in which individuals see and evaluate their own capabilities, especially with regard to the malleability of different attributes, such as intelligence and personality. 1 2

The mindset is divided into two approaches: fixed and growth. The growth mindset concerns the individual who believes that their capabilities are subject to change, according to their level of commitment, training and dedication. This type of belief is associated with the individual who is always willing to learn and, therefore, is prone to expanding their knowledge and skills. 1 2

On the other hand, those dominated by the fixed mindset, show themselves as individuals who believe that their ability is innate, hereditary, and not subject to improvement. These are those individuals most associated with the fear of failure, who limit themselves to the type of activity they know they already master, refusing new challenges. 1 2

Mindset theory began to be discussed in the 1970s. Carol S. Dweck highlighted the theory by analyzing the different ways in which children reacted when challenged to perform tasks of different levels of difficulty. And in 2007, an instrument was developed to evaluate individuals regarding their perception of their own intelligence. 1 2

With the advancement of studies and the dissemination of his ideas regarding mindset, 3 several adaptations emerged from the initial scale, making it possible to apply it not only to intelligence, but also to other areas of social and behavioral sciences, and, recently, being applied to the medical field. 3 4

The scale developed by Claudia Mueller et al. 4 provides a tool that can be validated in the national territory. It is an adaptation of the original scale by Dweck et al., 1 2 which consists of three items, with responses ranging from 1 (strongly disagree) to 6 (strongly agree) on the Likert scale 5 ( Table 1 ). In its initial publication, the scale was named the Health Belief Scale 6 and was later referred to as the Health Mindset Scale. 7

**Table 1:** Mindset Health Scale

1. Your body has a certain amount of health, and you really can't do much to change it					
1	2	3	4	5	6
Strongly agree			Strongly disagree		
2. Your health is something about you that you can't change very much					
1	2	3	4	5	6
Strongly agree			Strongly disagree		
3. You can try to make yourself feel better, but you can't really change your basic health					
1	2	3	4	5	6
Strongly agree			Strongly disagree		

In this way, expanding part of the concepts presented, the health-oriented mindset addresses how the individual perceives their own health, whether in a fixed, immutable, or growing way, having direct implications for the follow-up and treatment of this patient. Since the individual with a fixed mindset, in theory, believes that their health is innate, immutable, having little receptivity to health education and prevention policies, given that their health is not subject to change. 4 6 7

On the other hand, it is expected that the individual with a growth mindset will perceive their health as capable of improvement, being more open to health education, accepting new goals and challenges during therapeutic decisions, and, ultimately, being able to present greater quality of life. 4 6 7

## Materials e Methods

### Study design and ethical statements

The present questionnaire study for cross-cultural validation of the health-oriented mindset scale was developed between October 2021 and August 2022. The authors obtained formal authorization from the research group that developed the original questionnaire for application and validation of the scale in Brazilian territory. (Annex 1)

The study was approved by the Ethics and Research Committee on February 11, 2022. All caregivers of the study participants were duly informed and guided, receiving the Free and Informed Consent Form (FICF) for signature, with the appropriate specifications about the study, information about the researchers and role of the participant.

The study was carried out with patients from the Department of Orthopedics and Traumatology of our hospital. The sample consisted of 215 patients aged between 18 and 87 years (M = 41.98; SD = 15.72), the average age between 31 and 50 years (42.0%), and female (52 .6%), as detailed in Table 2 .

**Table 2:** Sample Characteristics

Variable	f	%
Sex		
Female	113	52,6
Male	102	47,4
Age range		
18 to 24 years old	22	10,2
25 to 30 years	43	20,0
31 to 40 years old	45	21,0
41 to 50 years old	45	21,0
51 to 60 years old	28	13,0
Over 60 years old	32	14,8

For cross-cultural adaptation of the scale, guidelines were used according to the method of Beaton et al. 8 .

Initially, the scale was translated from English (original language) to Brazilian Portuguese – the language of interest – independently by two native Brazilian individuals, not appearing as authors or co-authors of the work, one of them without medical training or knowledge, called “ Translator 1” and “Translator 2”. Both were native Brazilians and fluent in English.

Then, the two versions obtained were synthesized and reviewed by a third assessor, also fluent in both languages. For the purposes of resolving conflicts and standardizing the scale, a third scale was obtained, based on discussions between authors and translators, called “pre-test” ( Table 3 ).

**Table 3:** Pre-test scale

1. Seu corpo tem uma certa quantidade de saúde e não há muito que você possa fazer para mudá-la
2. Sua saúde é algo sobre você que não se pode mudar muito
3. Você pode tentar se sentir melhor, mas você realmente não pode mudar sua saúde básica

In this way, the “pre-test” scale was submitted to two bilingual translators, whose mother tongue was English, being fluent in the Brazilian Portuguese language, also without medical training or knowledge, highlighting that they did not know the topic, as well as not had knowledge about the original scale, carried out the reverse translation, that is, the “pre-test” scale was translated into English ( Table 4 ).

**Table 4:** Reverse translation

1. Your body has a certain amount of health and there isn't much you can do to change it
2. Your health is something that can't be changed very much
3. You can try to make yourself feel better, but your basic health caǹt be changed

An expert committee was formed, consisting of the translators, two orthopedic spine surgeons, and three general practitioners. This committee was responsible for reviewing the obtained translations. Through this analysis, it was possible to resolve issues of semantics, redundancy, and comprehension, resulting in the "pre-final" version of the health mindset scale in Brazilian Portuguese.

The "pre-final" scale was administered to 20 Brazilian patients under follow-up at the Spine Outpatient Clinic of the Department of Orthopedics and Traumatology at our hospital by the three authors of this study. During this stage, the interviewees were asked to select the best response that represented their understanding of the content. They were also encouraged to identify any questions or concerns regarding the clarity and comprehensibility of the presented questions, as well as any ambiguities.

Once again, the committee of experts was convened so that it could analyze the responses, in order to resolve conflicts, correct ambiguities and resolve and standardize the questions, as demonstrated in Table 5 .

**Table 5:** Understanding and translation process of the Health Mindset Scale

Question	Summary version		Final version	
	Discussion	Consensus	Discussion	Consensus
**1**	The expression “certain amount of health”	Grau de saúde	−	−
**2**	The expression “Your health is something about you”	Nossa saúde	The expression “change very much”	Mudar em quase nada
**3**	The expression “basic health”	Sua saúde	The expression “You can try to make yourself feel better”	Você tentar ter hábitos de vida mais saudáveis
**Answer**	Summary version		Final version	
	Discussion	Consensus	Discussion	Consensus
**Strongly agree**	Concordo fortemente	−	−	Concordo totalmente
**Strongly disagree**	Discordo fortemente	−	−	Discordo totalmente

In this way, the final version of the health mindset scale was obtained ( Table 6 ).

**Table 6:** Final Scale

1. O nível de saúde do nosso corpo já vem pronto, e isso a gente não pode mudar
2. Nossa saúde é algo que não podemos em quase nada
3. Você pode tentar ter hábitos de vida mais saudáveis, mas sua saúde não vai mudar muito

### Health Mindset Scale

The scale is made up of 03 questions, which seek to demonstrate how the individual perceives their own health, both due to innate health conditions and the possibility of improving or developing healthier lifestyle habits.

Responses are expressed through values on the Likert et al. scale, 5 ranging from 1 (totally disagree) to 6 (totally agree), according to Table 1

The individual is considered to have a growth mindset the closer their answers are to number 6 (I completely disagree), the opposite is also true: the closer the individual gets to number 1 in their answers, they will be considered to have a fixed mindset.

Internal consistency was assessed using Cronbach's alpha coefficient, 9 the purpose of which is to evaluate the homogeneity of differentiating factors between items that make up the same questionnaire or subgroups thereof. Therefore, a low coefficient indicates a lower correlation between items.

However, a high coefficient reflects redundancy between one or more items in the questionnaire. For this type of study, the recommended value for the coefficient should be between 0.70 and 0.95, in accordance with those observed by Terwee et al. 10

The internal consistency of the three items was satisfactory (α = 0.786). Table 7 presents descriptive statistics for each item and the α if the item is excluded.

**Table 7:** Descriptive statistics and α if the item is excluded from the Mindset Health Scale items

Item	M	SD	α if the item is deleted
1. O nível de saúde do nosso corpo já vem pronto, e isso a gente não pode mudar.	5,02	1,45	0,72
2. Nossa saúde é algo que não podemos mudar em quase nada.	5,32	1,29	0,70
3. Você pode tentar ter hábitos de vida mais saudáveis, mas sua saúde não vai mudar muito.	5,31	1,28	0,70

*Note: M = average; SD = standard deviation* .

Complementarily, Table 8 presents bivariate inter-item correlations.

**Table 8:** Inter-item, item-total and alpha correlations if the pain item excluded from the Mindset Health Scale

Item	1.	2.	3	Item-Total Correlation	α if the item is deleted
1. O nível de saúde do nosso corpo já vem pronto, e isso a gente não pode mudar.	1			0,62	0,72
2. Nossa saúde é algo que não podemos mudar em quase nada.	0,55	1		0,63	0,70
3. Você pode tentar ter hábitos de vida mais saudáveis, mas sua saúde não vai mudar muito.	0,54	0,56	1	0,63	0,70

High bivariate correlations were observed between items (r > 0.50). Item-total correlations ranged between 0.62 and 0.63. As shown in Table 7 , no item harms the total alpha value, given the estimate of α if the item is excluded.

## Results

### Sample Characteristics

The sample consisted of 215 patients aged between 18 and 87 years (M = 41.98; SD = 15.72), with a prevalence between 31 and 50 years (42.0%), and female (52.6 %), as demonstrated in Table 2 .

Cronbach's alpha coefficient was 0.75 for male patients and 0.80 for female patients. The results did not indicate a statistically significant difference for items 1 and 2. However, a marginally significant difference (p < 0.10) was observed between men and women for item 3. In this item, the average for men (M = 5 .48; SD = 0.99) was greater than the average for women (M = 5.10; SD = 1.22), with a small effect size for difference (d = 0.26) ( Table 9 ).

**Table 9:** Descriptive statistics and comparison by sex

	Male		Female		Difference		Effect Size
	M	SD	M	SD	t	p	Cohen's D
Item 1	5,12	1,39	4,92	1,51	1,04	0,297	0,13
Item 2	5,41	1,20	5,23	1,36	1,03	0,300	0,14
Item 3	5,48	1,05	5,15	1,44	1,90	0,054	0,26
Average total score	5,34	0,99	5,10	1,22	1,57	0,118	0,21

Additionally, the Pearson correlation between items and item-total correlation were presented. Correlations of magnitude were considered: weak - greater than 0.10; moderate - greater than 0.30; and strong - greater than 0.50. 11

Pearson's correlation coefficient “r” was examined between the mean score and age (r = −0.21; p = 0.002), the result of which indicated a weak, negative and significant linear relationship. The older the age, the lower the average score on the Health Mindset Scale.

Data analysis were carried out using the IBM SPSS 24.0 program. 12 The value of p < 0.05 was used because it was considered statistically significant and p < 0.10 was considered marginally significant.

### Differences by sex

Cronbach's alpha coefficient was 0.75 for male patients and 0.80 for female patients. Table 9 also presents the means for each item and the total mean by gender.

Aiming to compare item scores by sex, mean comparison statistics were performed using the T test for independent samples. The effect size of the difference between the sexes was investigated by calculating Cohen's D, 13 considering effect sizes: small (d > 0.20); moderate (d > 0.50); and strong (d > 0.80).

The overall results did not indicate a statistically significant difference. However, for item 3 there was a marginally significant difference (p < 0.10) between men and women. In this item, the mean for men (M = 5.48; SD = 0.99) was higher than the mean for women (M = 5.10; SD = 1.22), with a small effect size for the difference (d = 0.26).

### Descriptive statistics and floor and ceiling effect

Table 10 presents descriptive statistics (means and standard deviations) of the items, as well as the floor and ceiling effect.

**Table 10:** Descriptive statistics and floor and ceiling effects of Mindset Health Scale items

Item	M	SD	Floor - Ceiling
1. O nível de saúde do nosso corpo já vem pronto, e isso a gente não pode mudar.	5,02	1,45	6,5% - 57,2%
2. Nossa saúde é algo que não podemos mudar em quase nada.	5,32	1,29	4,2% - 68,8%
3. Você pode tentar ter hábitos de vida mais saudáveis, mas sua saúde não vai mudar muito.	5,31	1,28	5,1% - 65,1%

*Note: M = average; SD = standard deviation.*

According to the criteria established by McHorney 14 of 20% to indicate a floor and ceiling effect, it can be seen in Table 5 that the scale items show a considerable ceiling effect. There is a tendency for participants to select answer 6 on the scale. 57.2% of participants in item 1, 68.8% of participants in item 2, and 65.1% of participants in item 3 selected the highest point on the scale.

## Discussion

The questionnaire proposed by Dweck is based on two constructs: the growth mindset and the fixed mindset. Initially, the scale consisted of 8 questions, but a recurring complaint from interviewees was regarding the number of questions addressing the same topic, causing content redundancy, which was then reduced to 3, with good acceptance from participants. 4 6 7

It is important to initially highlight the scarcity of national work regarding the health-oriented mindset. The term mindset is widely discussed, derived from psychometric analysis as Carol S. Dweck described, 1 2 or as a synonym for behavioral programming, whether for entrepreneurship or to achieve a certain goal.

On the other hand, there are several works validating foreign scales using the Beaton method, 8 such as the cross-cultural validation of the Early-Onset Scoliosis 24 Item Questionnaire (EOSQ-24©) carried out by Mendonça. 15

The analysis of the health mindset opens up a range of possibilities for understanding the health-disease process in each patient, both through their vision of health and their adherence and engagement with therapeutic proposals and lifestyle changes. 16

In the case of healthy individuals, a constructive mindset is associated with better eating and physical activity habits, both in obese and eutrophic individuals. 16

In a study with 132 adolescents with type 1 diabetes, it was noted that patients with a growth mindset were more related to lower levels of glycosylated hemoglobin (HbA1c) and a greater frequency of self-tests throughout the day, both for younger patients. 17

Despite being a relatively new concept, the health mindset theory is very promising. Another study with 210 Native Americans suggested that patients with a growth mindset were more likely to adopt behaviors that could help reduce the transmission of COVID19 in community settings, adopting measures such as greater frequency of hand hygiene, use of alcohol gel and hygiene of personal items such as cell phones. 18

In addition to being an objective and quick-to-apply instrument, it can be easily analyzed in conjunction with other scales. It was correlated with the Scoliosis Research Society Health-Related Quality of Life (SRS-30) in a population of 110 adolescents, in which individuals with a growth mindset reported higher quality of life rates compared to patients with a fixed mindset. 19

Analyzing the mindset of parents and children seems to have a strong relationship with treatment results. It was noticed that parents with a growth mindset had more schooling, less emotional stress and fewer complaints of pain during follow-up and use of braces for adolescent idiopathic scoliosis, which led to less administration of analgesics. In this way, parents and patients with a growth mindset showed greater satisfaction with the treatment. 20

In chronic diseases, they present better glycemic control in diabetes 17 and better quality of life in the case of kidney transplant recipients. 21

In dialogue with the original authors of the scale, it was determined that it would not be possible to establish a specific cutoff point to categorize individuals as having either a fixed or growth mindset. The proximity to the extremes of the scale will determine where the patient falls on the spectrum.

Therefore, by identifying and intervening in patients or parents of patients with a fixed mindset, medical professionals and their teams have the potential to improve the quality of life for the patient through targeted approaches and more personalized treatments. When it comes to mindset, it relates to an individual's behavior, and interventions aimed at promoting a growth mindset can impact treatment adherence and potentially change the patient's outcome.

It was observed that the interviewees had a good understanding of the terms used, as the scale is quick and objective. Individuals under the age of 60 demonstrated a greater capacity for transitioning from a fixed mindset to a growth mindset. In other words, the younger the individual, the greater their potential for change. A properly directed treatment plan could potentially alter the patient's mindset, likely due to their life conditions resembling a growth mindset more closely.

## Conclusion

The Brazilian Portuguese version of the health mindset scale was presented and cross-culturally validated, showing a good reliability coefficient – Cronbach's alpha 0.786. Therefore, it truly constitutes a new tool for clinical practice, both globally and nationally. It is relevant both due to the wide range of possibilities for analyzing patient behavior, as well as the ease and speed of its application, as well as the possibility of correlating it with other scales.

Validation brought greater adaptation to Brazilian culture, in which no publications had yet been found, seeking greater efficiency when measuring the Mindset of individuals, aiming to achieve the objective for which the instrument proposes. And, above all, providing the assistant team with more tools for clinical practice and individualizing interventions and treatments to obtain better outcomes during medical practice.

To correlate with other instruments, it is suggested to continue the study, initially in an exploratory manner, leading to a confirmatory study with a larger sample, making the results more generalizable.
