# Supplementary material for: Cross-cultural Validation of the Health Mindset Scale for Brazil
Source: Rev Bras Ortop (Sao Paulo). 2024 Apr 10;59(2):e213–9. doi: 10.1055/s-0044-1779329 (PMC11006518; doi:10.1055/s-0044-1779329)
Supplement: Related texts

# Research article: Validação transcultural da Escala de *Mindset* Direcionado à Saúde no Brasil

**Keywords:** qualidade de vida, inquéritos e questionários, reprodutibilidade dos testes, saúde

## Abstract

**Objetivo** Validar transculturalmente a escala de *mindset* da saúde para o Brasil, bem como adaptar os termos e questionários para a adequada compreensão do brasileiro, utilizando a análise fatorial como instrumento para validar a sua confiabilidade.

**Métodos** Validação transcultural da *Health Mindset Scale* para o português brasileiro através do método do Beaton, cálculo do alfa de *Cronbach* e análise fatorial

**Resultados** Amostra foi composta por 215 pacientes com idades entre 18 e 87 anos (M = 41,98; DP = 15,72), sendo a média de idade entre 31 e 50 anos (42,0%), e do sexo feminino (52,6%), uma diferença marginalmente significativa (p < 0,10) entre homens e mulheres foi observada para o item 3. Nesse item a média dos homens (M = 5,48; DP = 0,99) foi maior do que a média das mulheres (M = 5,10; DP = 1,22), com tamanho de efeito para diferença pequeno (d = 0,26). O coeficiente de correlação r de Pearson foi examinado entre o escore médio e a idade ( *r* = −0,21; *p* = 0,002), cujo resultado indicou relação linear fraca, negativa e significativa. Quanto maior a idade, menor o escore médio na *Health Mindset Scale.*

**Conclusão** A versão da escala de *mindset* direcionada à saúde para o português brasileiro foi apresentada e validade transculturalmente, apresentando bom coeficiente de confiabilidade – alfa de *Cronbach* 0,786. Sendo assim, configura novo instrumento para a prática clínica e pode ser correlacionado escalas já consagradas na literatura.

## Introdução

A teoria do *mindset* propõe as formas pelas quais o indivíduo enxerga e avalia as suas próprias capacidades, principalmente no que diz respeito a maleabilidade de atributos diversos, como inteligência e personalidade. 1 2

O *mindset* é dividido sobre dois enfoques: o fixo e o de crescimento. O *mindset* de crescimento diz respeito ao indivíduo que acredita que suas capacidades são passíveis de mudança, de acordo com o seu grau de empenho, treino e dedicação. Esse tipo de crença está associado ao indivíduo que sempre está disposto a aprender e, sendo assim, está propenso a expandir o seu conhecimento e suas habilidades. 1 2

Por outro lado, aqueles dominados pelo *mindset* fixo, se mostram como indivíduos que acreditam que sua capacidade é inata, hereditária, não sendo passível de aperfeiçoamento. São aqueles indivíduos mais associados ao medo de fracassar, que se limitam ao tipo de atividade que sabidamente já dominam, recusando novos desafios. 1 2

A teoria do *mindset* começou a ser discutida na década de 70. Carol S. Dweck trouxe destaque para a teoria ao analisar as diferentes formas que as crianças reagiam ao serem desafiadas a realizar tarefas de diferentes níveis de dificuldades. E em 2007 foi elaborado instrumento que avaliasse o indivíduo quanto a sua percepção da própria inteligência. 1 2

Com o avançar dos estudos e a divulgação de suas ideias quanto ao *mindset* , 3 diversas adaptações surgiram a partir da escala inicial, sendo possível a sua aplicação não somente a inteligência, mas também para outras áreas das ciências sociais e comportamentais, e, recentemente, sendo aplicada para a área médica. 3 4

Tem-se na escala desenvolvida por Claudia Mueller et al. 4 a ferramenta passível de validação no território nacional. Trata-se de adaptação da escala original de Dweck et al., 1 2 a qual possui três quesitos, cujas respostas compreendem valores da escala de Likert et al., 5 variando de 1 (discordo totalmente) a 6 (concordo totalmente) ( Tabela 1 ). Em sua primeira publicação, a escala foi denominada *Health Belief Scale* , 6 sendo denominada posteriormente *Health Mindset Scale* . 7

**Tabela 1:** *Mindset Health Scale*

1. Your body has a certain amount of health, and you really can't do much to change it					
1	2	3	4	5	6
Strongly agree			Strongly disagree		
2. Your health is something about you that you can't change very much					
1	2	3	4	5	6
Strongly agree			Strongly disagree		
3. You can try to make yourself feel better, but you can't really change your basic health					
1	2	3	4	5	6
Strongly agree			Strongly disagree		

Desta forma, ampliando parte dos conceitos apresentados, o *mindset* direcionado à saúde aborda como o indivíduo percebe a própria saúde, seja de forma fixa, imutável, ou de crescimento. Tendo implicações diretas no seguimento e tratamento deste paciente. Uma vez que o indivíduo de *mindset* fixo, em tese, acredita que sua saúde é inata, imutável, tendo pouca receptividade quanto a educação em saúde e políticas de prevenção, haja vista a sua saúde não ser passível de mudanças. 4 6 7

Por outro lado, espera-se que o indivíduo do *mindset* de crescimento perceberá a saúde como passível de aperfeiçoamento, sendo mais aberto a educação em saúde, aceitando novas metas e desafios durante as decisões terapêuticas, bem como, em última análise, poderá apresentar maior qualidade de vida. 4 6 7

## Materiais e Métodos

## Desenho do estudo e afirmações éticas

O presente estudo de questionário para validação transcultural da escala de *mindset* direcionado à saúde foi desenvolvido entre outubro de 2021 e agosto de 2022. Os autores obtiveram autorização formal do grupo de pesquisa desenvolvedor do questionário original para aplicação e validação da escala no território brasileiro. (Anexo 1)

O estudo foi aprovado pelo Comitê̂ de Ética e Pesquisa em 11 de fevereiro de 2022. Todos os cuidadores dos participantes do estudo foram informados e orientados devidamente, recebendo para assinatura o Termo de Consentimento Livre e Esclarecido (TCLE), com as devidas especificações sobre o estudo, informação sobre os pesquisadores e papel do participante.

O estudo foi realizado com pacientes do Departamento de Ortopedia e Traumatologia do nosso hospital. A amostra foi composta por 215 pacientes com idades entre 18 e 87 anos (M = 41,98; DP = 15,72), a média de idade entre 31 e 50 anos (42,0%), e do sexo feminino (52,6%), como detalha a Tabela 2 .

**Tabela 2:** Perfil da amostra

Variável	f	%
**Sexo**		
Feminino	113	52,6
Masculino	102	47,4
**Faixa etária**		
18 a 24 anos	22	10,2
25 a 30 anos	43	20,0
31 a 40 anos	45	21,0
41 a 50 anos	45	21,0
51 a 60 anos	28	13,0
Acima de 60 anos	32	14,8

Para adaptação transcultural da escala foram utilizadas as orientações segundo o método de Beaton et al. 8

Inicialmente, a escala foi traduzida do inglês (língua original) para o português brasileiro – língua de interesse – de maneira independente por dois indivíduos brasileiros natos, não figurando como autores ou coautores do trabalho, sendo um deles sem formação ou conhecimento médico, denominados “Tradutor 1” e “Tradutor 2”. Ambos eram brasileiros natos e fluentes em inglês.

Em seguida, as duas versões obtidas foram sintetizadas e revisadas por um terceiro assessor, também fluente em ambos os idiomas. Para efeitos de resolução de conflitos e uniformização da escala, foi obtida uma terceira escala, a partir de discussões entre autores e tradutores, denominada “pré-teste” ( Tabela 3 ).

**Tabela 3:** Escala pré-teste

1. Seu corpo tem uma certa quantidade de saúde e não há muito que você possa fazer para mudá-la
2. Sua saúde é algo sobre você que não se pode mudar muito
3. Você pode tentar se sentir melhor, mas você realmente não pode mudar sua saúde básica

Desta forma, a escala “pré-teste” foi submetida a dois tradutores bilingues, cuja língua materna era a inglesa, sendo fluentes no idioma português brasileiro, também sem formação ou conhecimento médico, destacando-se que não conheciam o tema, bem como não possuíam conhecimentos sobre a escala original, realizaram a tradução reversa, ou seja, a escala “pré-teste” foi traduzida para o inglês ( Tabela 4 ).

**Tabela 4:** Tradução reversa

1. Your body has a certain amount of health and there isn't much you can do to change it
2. Your health is something that can't be changed very much
3. You can try to make yourself feel better, but your basic health caǹt be changed

Foi formado um comitê de especialistas, composto pelos tradutores, dois cirurgiões ortopédicos de coluna vertebral e três médicos generalistas, o qual ficou responsável pela revisão das traduções obtidas. A partir desta análise, foi possível dirimir conflitos de semântica, de redundância e compreensão, configurando a versão “pré-final” da escala de *mindset* direcionado à saúde para o português brasileiro.

A escala “pré-final” foi aplicada em 20 pacientes brasileiros em seguimento no ambulatório de Coluna do Departamento de Ortopedia e Traumatologia do nosso hospital pelos três assistentes deste trabalho.

Nesta etapa, os entrevistados deveriam assinalar a melhor resposta que representasse a compreensão do que fora posto. Também foi solicitado aos entrevistados que apontassem eventuais dúvidas sobre as questões, tanto quanto a compreensão, quanto ambiguidades e clareza do que fora exposto.

Novamente o comitê de especialistas foi convocado para que pudesse analisar as respostas, de modo a dirimir conflitos, corrigir ambiguidades e sanar e padronizar os quesitos, como demonstrado na Tabela 5 .

**Tabela 5:** Compreensão e processo de tradução da *Health Mindset Scale*

Questão	Versão de síntese		Versão final	
	Discussão	Consenso	Discussão	Consenso
**1**	The expression “certain amount of health”	Grau de saúde	−	−
**2**	The expression “Your health is something about you”	Nossa saúde	The expression “change very much”	Mudar em quase nada
**3**	The expression “basic health	Sua saúde	The expression “You can try to make yourself feel better”	Você tentar ter hábitos de vida mais saudáveis
**Respostas**	Versão de síntese		Versão final	
	Discussão	Consenso	Discussão	Consenso
**Strongly agree**	Concordo fortemente	−	−	Concordo totalmente
**Strongly disagree**	Discordo fortemente	−	−	Discordo totalmente

Desta forma, foi obtida a versão final da Escala de *Mindset* Direcionado à Saúde ( Tabela 6 ).

**Tabela 6:** Escala final

1. O nível de saúde do nosso corpo já vem pronto, e isso a gente não pode mudar
2. Nossa saúde é algo que não podemos em quase nada
3. Você pode tentar ter hábitos de vida mais saudáveis, mas sua saúde não vai mudar muito

## Escala de *Mindset* Direcionado À Saúde

A escala é composta por 03 quesitos, que buscam demonstrar como o indivíduo percebe a própria saúde, tanto pelas condições inatas da saúde quanto pela possibilidade de melhorar ou de desenvolver hábitos de vida mais saudáveis.

As respostas são expressadas através de valores da escala de Likert et al., 5 variando de 1 (discordo totalmente) a 6 (concordo totalmente), conforme Tabela 1

O indivíduo é tido como portador do *mindset* de crescimento quanto mais suas respostas se aproximarem do número 6 (discordo totalmente), o contrário também é verdadeiro, quanto mais o indivíduo se aproximar do número 1 nas suas respostas, será considerado como portador do *mindset* fixo.

A consistência interna foi avaliada pelo coeficiente alfa de *Cronbach* , 9 cuja finalidade é avaliar a homogeneidade dos fatores de diferenciação entre itens que componham o mesmo questionário ou subgrupos deste. Desta forma baixo coeficiente indica menor correlação entre itens.

No entanto, alto coeficiente traduz redundância entre um ou mais itens do questionário. Para este tipo de estudo o valor recomendado para o coeficiente deverá encontrar-se entre 0,70 e 0,95, de acordo com os observado por Terwee et al. 10

A consistência interna dos três itens foi satisfatória (α = 0,786). A Tabela 7 apresenta estatísticas descritivas de cada item e o α se o item for excluído.

**Tabela 7:** Estatísticas descritivas e α se o item for excluído dos itens da *Mindset Health Scale*

Item	M	DP	α se o item for excluído
1. O nível de saúde do nosso corpo já vem pronto, e isso a gente não pode mudar.	5,02	1,45	0,72
2. Nossa saúde é algo que não podemos mudar em quase nada.	5,32	1,29	0,70
3. Você pode tentar ter hábitos de vida mais saudáveis, mas sua saúde não vai mudar muito.	5,31	1,28	0,70

*Nota* : M = média; DP = desvio-padrão.

De forma complementar, a Tabela 8 apresenta correlações bivariadas inter-itens.

**Tabela 8:** Correlações inter-itens, item-total e alfa se o item dor excluído da *Mindset Health Scale*

Item	1	2	3	Correlação Item-Total	α se o item for excluído
1. O nível de saúde do nosso corpo já vem pronto, e isso a gente não pode mudar.	1			0,62	0,72
2. Nossa saúde é algo que não podemos mudar em quase nada.	0,55	1		0,63	0,70
3. Você pode tentar ter hábitos de vida mais saudáveis, mas sua saúde não vai mudar muito.	0,54	0,56	1	0,63	0,70

Correlações bivariadas altas foram observadas entre os itens (r > 0,50). As correlações item-total variaram entre 0,62 e 0,63. Como apresentado na Tabela 7 , nenhum item prejudica o valor de alfa total, dada a estimativa do α se o item for excluído.

## Resultados

## Características da Amostra

A amostra foi composta por 215 pacientes com idades entre 18 e 87 anos (M = 41,98; DP = 15,72), com prevalência entre 31 e 50 anos (42,0%), e do sexo feminino (52,6%), como demonstrado na Tabela 2 .

O coeficiente alfa de *Cronbach* foi 0,75 para pacientes do sexo masculino e 0,80 para pacientes do sexo feminino. Os resultados não indicaram diferença estatisticamente significativa para os itens 1 e 2. No entanto, foi notada diferença marginalmente significativa (p < 0,10) entre homens e mulheres foi observada para o item 3. Nesse item a média dos homens (M = 5,48; DP = 0,99) foi maior do que a média das mulheres (M = 5,10; DP = 1,22), com um tamanho de efeito para diferença pequeno (d = 0,26) ( Tabela 9 ).

**Tabela 9:** Estatísticas descritivas e comparação por sexo

	Masculino		Feminino		Diferença		Tamanho de efeito
	M	DP	M	DP	t	p	d de Cohen
Item 1	5,12	1,39	4,92	1,51	1,04	0,297	0,13
Item 2	5,41	1,20	5,23	1,36	1,03	0,300	0,14
Item 3	5,48	1,05	5,15	1,44	1,90	0,054	0,26
Escore total médio	5,34	0,99	5,10	1,22	1,57	0,118	0,21

Adicionalmente, apresentou-se a correlação de Pearson entre itens e correlação item-total. Foram consideradas correlações de magnitude: fraca - superiores a 0,10; moderadas - superiores de 0,30; e fortes - superiores a 0,50. 11

O coeficiente de correlação *“r”* de Pearson foi examinado entre o escore médio e a idade ( *r* = −0,21; *p* = 0,002), cujo resultado indicou relação linear fraca, negativa e significativa. Quanto maior a idade, menor o escore médio na *Health Mindset Scale.*

As análises de dados foram realizadas utilizando o programa o IBM SPSS 24.0. 12 O valor de p < 0,05 foi utilizado por ser considerado estatisticamente significativo e p < 0,10 foi considerado como marginalmente significativo.

## Diferenças por sexo

O coeficiente alfa de *Cronbach* foi 0,75 para pacientes do sexo masculino e 0,80 para pacientes do sexo feminino. A Tabela 9 também apresenta as médias de cada item e a média total por sexo.

Visando um comparativo das pontuações dos itens por sexo, foram realizadas estatísticas de comparação de médias por meio do teste T para amostras independentes. O tamanho do efeito da diferença entre os sexos foi investigado pelo cálculo do D de Cohen, 13 considerando tamanhos de efeito: pequeno (d > 0,20); moderado (d > 0,50); e forte (d > 0,80).

Os resultados gerais não indicaram diferença estatisticamente significativa. No entanto, para o item 3 houve uma diferença marginalmente significativa (p < 0,10) entre homens e mulheres. Nesse item a média dos homens (M = 5,48; DP = 0,99) foi maior do que a média das mulheres (M = 5,10; DP = 1,22), com um tamanho de efeito para diferença pequeno (d = 0,26).

## Estatísticas descritivas e Efeito Chão e Teto

A Tabela 10 apresenta estatísticas descritivas (médias e desvios padrão) dos itens, bem como o efeito de chão e teto.

**Tabela 10:** Estatísticas descritivas e efeito chão e teto dos itens da Mindset Health Scale

**Item**	**M**	**DP**	**Chão – teto**
1. O nível de saúde do nosso corpo já vem pronto, e isso a gente não pode mudar.	5,02	1,45	6,5% - 57,2%
2. Nossa saúde é algo que não podemos mudar em quase nada.	5,32	1,29	4,2% - 68,8%
3. Você pode tentar ter hábitos de vida mais saudáveis, mas sua saúde não vai mudar muito.	5,31	1,28	5,1% - 65,1%

*Nota* : M = média; DP = desvio padrão.

De acordo com os critérios estabelecidos por McHorney 14 de 20% para indicativo de efeito de chão e teto, observa-se na Tabela 5 que os itens da escala evidenciam considerável efeito de teto. Há uma tendência dos participantes em selecionar a resposta 6 da escala. Selecionaram o maior ponto da escala 57,2% dos participantes no item 1, 68,8% dos participantes no item 2, e 65,1% dos participantes no item 3.

## Discussão

O questionário proposto por Dweck baseia-se em dois construtos: o *mindset* de crescimento e o *mindset* fixo. Inicialmente a escala era composta por 8 quesitos, porém uma queixa recorrente dos entrevistados era referente ao número de questões abordando uma mesma temática, causando redundância de conteúdo, sendo então reduzido para 3, com boa aceitação dos participantes. 4 6 7

Importante destacar inicialmente a escassez de trabalhos nacionais no tocante ao *mindset* direcionado a saúde. Discute-se muito o termo *mindset* , derivado das análises psicométricas como a Carol S. Dweck descreveu, 1 2 ou então como sinônimo de programação comportamental, seja para o empreendedorismo ou para alcançar determinado fim.

Encontra-se, em contrapartida, diversos trabalhos de validação de escalas estrangeiras pelo método de Beaton, 8 como na validação transcultural da *Early-Onset Scoliosis 24 Item Questionnaire* ” (EOSQ-24©) realizada por Mendonça. 15

A análise do *mindset* para a saúde abre um leque de possibilidades para a compreensão processo saúde-doença em cada paciente, tanto pela sua visão da saúde, quanto pela sua adesão e engajamento às propostas terapêuticas e mudança de estilo de vida. 16

No caso de indivíduos saudáveis, a mentalidade construtiva está associada a melhores hábitos de alimentação e de atividade física, tanto em obesos quanto em eutróficos. 16

Em um estudo com 132 adolescentes portadores de diabetes tipo 1, notou-se que pacientes com *mindset* de crescimento estavam mais relacionados a menores índices de hemoglobina glicosilada (HbA1c) e a maior frequência de autotestes ao longo do dia, ambos para pacientes mais jovens. 17

Apesar de conceito relativamente novo, a teoria do *mindset* direcionado à saúde é bastante promissora, em outro estudo com 210 índios americanos sugeriu que pacientes portadores de *mindset* de crescimento estavam mais associados a adotar comportamentos que poderiam ajudar a reduzir a transmissão da COVID19 em comunidades comunitárias, adotando medidas como maior frequência de higienização das mãos, uso de álcool em gel e higienização de itens pessoais como celulares. 18

Além de tratar-se de instrumento objetivo e de rápida aplicação, pode ser facilmente analisado em conjunto com outras escalas. Foi correlacionada com o *Scoliosis Research Society Health-Related Quality of Life* (SRS-30) em uma população de 110 adolescentes, nos quais os indivíduos com *mindset* de crescimento relatavam índices maiores de qualidade de vida em comparação aos pacientes com *mindset* fixo. 19

A análise do *mindset* de pais e filhos parece ter forte relação com os resultados do tratamento. Percebeu-se que pais com *mindset* de crescimento possuíam mais tempo de escolaridade, menos estresse emocional e menor quantidade de queixas de dor durante o seguimento e uso do colete para escoliose idiopática do adolescente, o que gerava menor administração de analgésicos. Desta forma, pais e pacientes com *mindset* do crescimento apresentaram maior satisfação com o tratamento. 20

Nas doenças crônicas, apresentam melhores controles glicêmicos na diabetes 17 e mais qualidade de vida no caso de transplantados renais. 21

Em diálogo com os autores originais da escala, foi informado que não seria possível estabelecer um ponto de corte a partir do qual se consideraria o indivíduo propenso ao *mindset* fixo ou de crescimento. A maior proximidade com os extremos da escala ditará em qual espectro o paciente se encaixará.

Desta forma, uma vez que o médico e a equipe assistente sejam capazes de identificar e intervir em pacientes ou pais de pacientes com *mindset* fixo, nós teremos o potencial de melhorar a qualidade de vida do doente, com abordagens direcionadas e tratamentos mais individualizados. No mesmo sentido, ao tratar-se de *mindset* , trata-se de comportamento do indivíduo, sendo assim, intervenções de *mindset* de crescimento podem ter o condão de afetar a adesão ao tratamento e mudar o desfecho do doente.

Foi possível avaliar que os entrevistados obtiveram boa compreensão dos termos utilizados, afinal, trata-se de escala rápida e objetiva. Os entrevistados com idade abaixo de 60 anos, apresentam maior capacidade de mudança do *mindset* fixo para o de crescimento, ou seja, quanto menor a idade, maior sua capacidade de mudança. O plano terapêutico adequadamente direcionado seria capaz de mudar o padrão do paciente, possivelmente devido suas condições vida que se assemelhem mais com o *mindset* de crescimento.

## Conclusão

A versão da escala de *mindset* direcionada à saúde para o português brasileiro foi apresentada e validada transculturalmente, apresentando um bom coeficiente de confiabilidade – alfa de *Cronbach* 0,786. Sendo assim, configura realmente um novo instrumento para a prática clínica, tanto a nível mundial, quanto nacional. Tendo sua relevância tanto pelo vasto leque de possibilidades de análises de comportamento dos pacientes, quanto pela facilidade e rapidez de sua aplicação, bem como pela possibilidade de correlacionar com outras escalas.

A validação trouxe maior adequação à cultura brasileira, na qual ainda não havia sido encontrado publicações, buscando uma maior eficiência ao medir o *Mindset* de indivíduos, visando atingir com primazia o objetivo no qual o instrumento se propõe. E, acima de tudo, fornecer a equipe assistente mais ferramentas para a prática clínica e, individualizando intervenções e tratamentos para obter melhores desfechos durante a prática médica.

Para correlacionar outros instrumentos, sugere-se a continuação do estudo, inicialmente com caráter exploratório, para um estudo confirmatório, com uma amostra maior e, assim, mais generalizável.
